# A note on the catch-up time method for estimating lead or sojourn time in prostate cancer screening

**DOI:** 10.1002/sim.5750

**Published:** 2013-01-31

**Authors:** Gerrit Draisma, Joost Rosmalen

**Affiliations:** a Department of Public Health, Erasmus MCP.O. Box 2040 3000, CA Rotterdam, The Netherlands; b Department of Biostatistics, Erasmus MCP.O. Box 2040 3000, CA Rotterdam, The Netherlands

**Keywords:** cancer screening, prostate cancer, lead time, sojourn time

## Abstract

Models of cancer screening assume that cancers are detectable by screening before being diagnosed clinically through symptoms. The duration of this preclinical phase is called sojourn time, and it determines how much diagnosis might be advanced in time by the screening test (lead time). In the catch-up time method, mean sojourn time or lead time are estimated as the time needed for cumulative incidence in an unscreened population to catch up with the detection rate (prevalence) at a first screening test. The method has been proposed as a substitute of the prevalence/incidence ratio in the case of prostate cancer where incidence cannot be treated as a constant. A model is proposed to justify this estimator. It is shown that this model is different from classic Markov-type models developed for breast cancer screening. In both models, the catch-up time method results in biased estimates of mean sojourn time. Copyright © 2013 John Wiley & Sons, Ltd.

## 1. Introduction

The objective of cancer screening is to detect tumors in an early stage with better chances of success of curative treatment. Since the randomized trials of breast cancer screening in the seventies and eighties and the introduction of breast cancer and cervical cancer screening, authors have developed statistical models for the evaluation of screening programs 1, 2. In these models, clinical diagnosis of cancer (e.g., due to symptoms) is preceded by a preclinical phase in which cancer is detectable with a suitable screening test, the so-called preclinical detectable phase (PCDP). The duration of the PCDP is known as sojourn time, and the time by which screening advances diagnosis, that is, the time between detection by screening and diagnosis in the absence of screening, is known as lead time. In a way, mean sojourn time and mean lead time measure the quality of a screening test, by indicating how much earlier tumors can be detected by the test.

Sojourn time and lead time are unobserved quantities, but mean values can be estimated by comparing the detection rate at (first) screening and cancer incidence in the absence of screening. A common estimate is the so-called prevalence/incidence ratio: based on a long-known relation between prevalence of preclinical disease (i.e., the probability *P* of the presence of preclinical cancer), incidence *I*, and mean sojourn time *μ*:


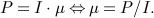
(1)

Prevalence is estimated from the detection rate *R* at first screening and the sensitivity of the screening test *s*: *P* = *R* / *s*. This model may be adequate when sojourn times are short and incidence can be treated as a constant, for instance, in the case of breast cancer with an estimated mean sojourn time of 1–2 years 1, 2. However, in the case of prostate cancer, *P* / *I* ratios of 8–12 years have been reported, and with incidence increasing with age and/or time, it cannot be treated as a constant. For the case of exponentially distributed sojourn times, Zelen and Feinleib 1 showed that the relation still holds for prevalence and incidence at the time of screening.

**Figure 1:**
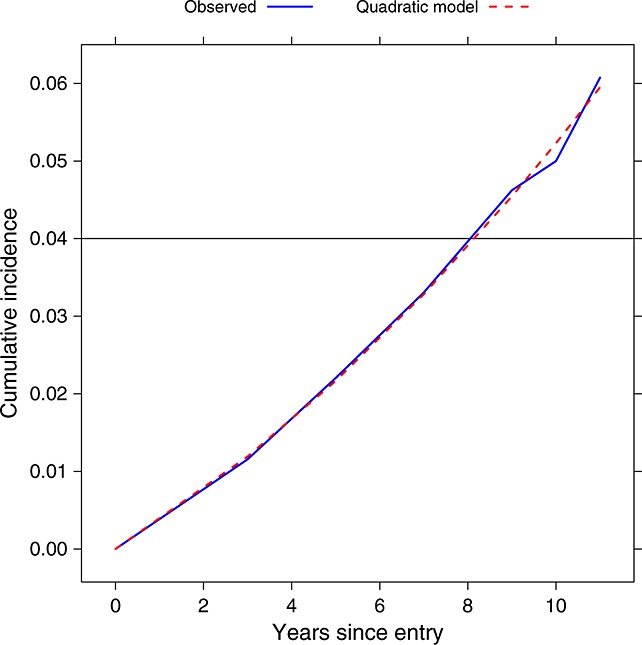
The catch-up time estimate for mean sojourn time. The graph shows the cumulative incidence of prostate cancer in the control arm from study entry in the Rotterdam section of ERSPC. The horizontal line represents the prevalence (4%) of preclinical prostate cancer at the first screening test. Cumulative incidence in the control arm catches up with prevalence after 8.16 years. The dashed line represents the quadratic approximation of cumulative incidence, 0.0034342704*x* + 0.0001796227*x*^2^ used in the numerical example. Figure adapted from Finne *et al.* 4.

Recently, several authors 3–6 have used the catch-up time method for estimating mean sojourn time, where mean sojourn time is estimated as the time needed for the cumulative incidence in an unscreened population to catch up with the detection rate in the first round of screening. Figure 1, reproduced from 4, illustrates the estimation for the Rotterdam section of the European Randomized Study of Screening for Prostate Cancer (ERSPC). Cumulative incidence in the control arm takes 8.16 years to reach the detection rate of 4.0% in the first round of screening in the screening arm.

The relation between the sojourn time and lead time distributions is not straightforward, except in the case of exponentially distributed sojourn times. In that case, the hazard of clinical diagnosis, marking the end of the sojourn time, is constant. The remaining time from detection by screening to clinical diagnosis is again exponentially distributed with the same hazard rate. In practical applications, exponentially distributed sojourn times are commonly assumed.

In the following, we first present a simple statistical model behind the catch-up time method, then show that in this model, the catch-up time method results in a biased sojourn time estimate, and finally, we show how this model differs from the classic models of Zelen and Feinleib 1 and Walter and Day 2. The experience of prostate cancer screening in the Rotterdam Center is worked out numerically to illustrate the effect of estimation methods on the estimated mean sojourn time. We also compare our results with the maximum-likelihood methods and estimates of these authors for breast cancer screening.

## 2. A model for the catch-up time method

**Figure 2:**
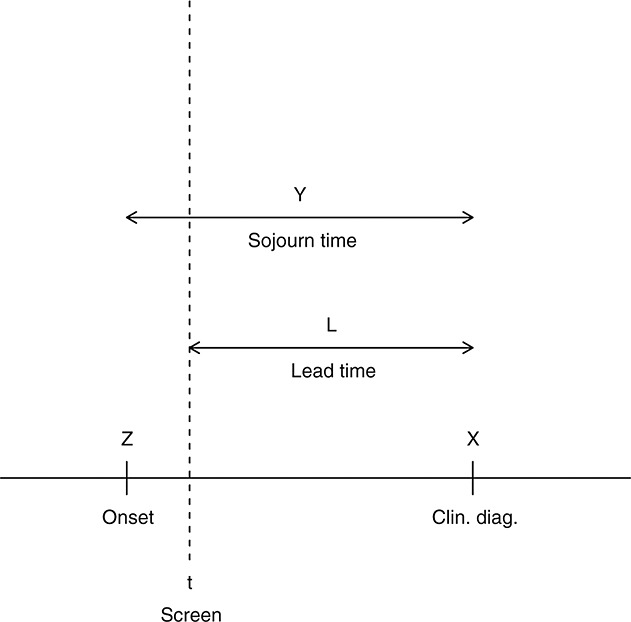
Relevant variables in screening models. Screening at time *t* may detect cancers with time of clinical diagnosis *X* > *t* and sojourn time *Y* > *X* − *t*, that is, with onset of preclinical disease *Z* < *t*. In case of detection, diagnosis has been advanced with lead time *L*.

Figure 2 shows the relevant random variables: Let *X* be the time of clinical diagnosis, in the absence of screening, *Y* the duration of the PCDP, and *Z* the time of onset of the preclinical phase. Assume that a screening test is taken at time *t*. Let *f*(*x*,*y*) be the joint distribution density of *X* and *Y*. Prevalent cancers at time *t* are cancers to be diagnosed at some time *X* > *t* with a sojourn time *Y* > *X* − *t* or equivalently, cancers with sojourn time *Y*, to be diagnosed at some time *X* > *t* such that *X* − *t ≤ Y*. The prevalence (the probability to be in the PCDP) at time *t* is given by


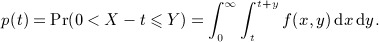
(2)

Here is the link with cumulative incidence: Pr(0 < *X* − *t ≤ Y* ) is the cumulative incidence from time of screening *t* to *t* + *Y*.

A first simplification is to assume independence between time of clinical diagnosis and sojourn time, that is, assuming *f*(*x*,*y*) = *I*(*x*) × *f*(*y*), where *I*(*x*) is the incidence at time *x* and *f*(*y*) the sojourn time density distribution. We shall refer to this model as the catch-up time model. In this model, we have


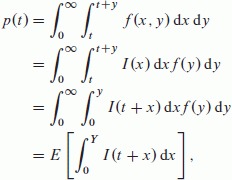
(3)

Equation (3) shows the relation between prevalence, cumulative incidence from time *t*, and the sojourn time distribution: Prevalence at time *t* equals the expected value of cumulative incidence up to time *Y* from time *t*.

However, this is not quite the same as the assumption made in the catch-up time method. There, it is assumed that mean sojourn time *E*(*Y* ) = *μ* equals the time needed for cumulative incidence to catch up with prevalence, that is,


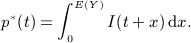
(4)

Comparing the right-hand side of these shows that (3) equates prevalence and mean (expected) cumulative incidence at sojourn time *Y*, and (4) equates prevalence and cumulative incidence at mean (expected) sojourn time *E*(*Y* ). Unfortunately, the mean of a function of a random variable is not generally equal to the value of that function at the mean of the variable, unless the function is linear, in our case when incidence is constant, *I*(*t*) = *I*. In fact, a famous inequality in statistics, Jensen's inequality, states that, given a random variable *Y* and a convex function *g*, the expectation of the random variable *g*(*Y* ) is larger than the value of *g* evaluated in the expected value of *Y*:


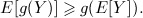
(5)

When incidence is increasing with time, cumulative incidence 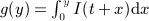 is a strictly convex function of *y* and Jensen's inequality (5) applies and so p*(*t*) < *p*(*t*). As a consequence, in this model, estimates of mean sojourn time based on equating p*(*t*) in Equation (4) to observed prevalence are too large.

## 3. A numerical example

Let us take the case of ERSPC-Rotterdam as given by Finne 4 as an example (Figure 1). Assume a quadratic function *β*_1_*x* + (*β*_2_ / 2)*x*^2^ for cumulative incidence as a function of time since study entry *x* and an exponential distribution with mean *μ*, that is, density *f*(*y*) = (1 / *μ*)*e* ^− *y* / *μ*^, for sojourn time. Then substituting 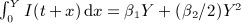 in (3) and using *E*(*Y* ) = *μ* and *E*(*Y*^2^) = 2*μ*^2^ for the exponential distribution, we have


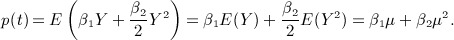
(6)

Solving this quadratic equation and substituting the values of Figure 1 results in an estimated mean sojourn time of 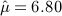 years. The catch-up time method proposes to substitute *E*(*Y* ) in the cumulative incidence function and solve


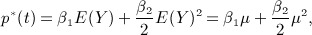
(7)

giving 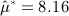 years.

By the way, this numerical example nicely shows that, when assuming a polynomial expression for (cumulative) incidence, prevalence can be expressed in terms of moments of the sojourn time distribution.

## 4. Classic screening models

In our catch-up time model, Equation (3), we assumed independence of clinical incidence and sojourn time (*X* and *Y* in Figure 2). In the classic screening models 1, 2, reasoning starts with the incidence of preclinical disease, assuming independence between sojourn time and preclinical incidence (*Y* and *Z* in Figure 2). Let *I*_*o*_ be the incidence of preclinical disease (onset). In this model, we have


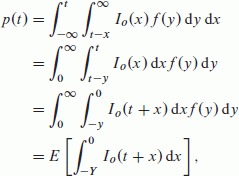
(8)

showing prevalence to be equal to the mean cumulative incidence of preclinical disease, cumulated from time of screening minus sojourn time up to time of screening. Unfortunately, preclinical incidence cannot be observed, but it can be computed from clinical incidence and the sojourn time distribution. This involves solving a so-called Volterra or convolution equation


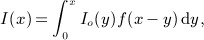
(9)

as proposed for instance by Pinsky 7.

Note that in this classic model, clinical incidence and sojourn time are no longer independent. But the conditional incidence given the sojourn time *I*(*x* | *y*) equals *I*_*o*_(*x* − *y*). It follows that 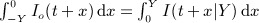, the conditional cumulative clinical incidence given sojourn time. That is, Equation (8) is Equation (3) with incidence *I*(*t* + *x*) replaced by the conditional incidence *I*(*t* + *x* | *y*).

## 5. Numerical example continued

If the distribution of sojourn time is exponential with density *f*(*y*) = (1 / *μ*)*e* ^− *y* / *μ*^, then (9) has the following solution:


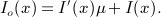


In our numerical example, with screening at time *t*, we have


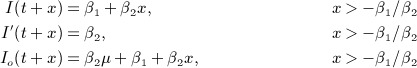


and


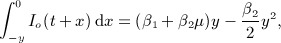
(10)

a *concave* function in *y*. Solving


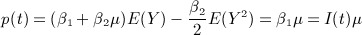
(11)

gives 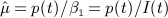 (11.64 years). Of course, this result was already proven by Zelen and Feinleib in 1969 1: In the case of exponentially distributed sojourn times, we have *I*(*t*) = *p*(*t*) / *μ*. One may note that we have been a bit careless by ignoring the condition *x* > − *β*_1_ / *β*_2_ or *y* < *β*_1_ / *β*_2_ in Equations (10) and (11), but prevalence *p*(*t*) = *I*(*t*)*μ* remains valid as shown by Zelen and Feinleib. Interestingly, the catch-up time method applied to the onset incidence *I*_*o*_ before the time of screening, that is, solving


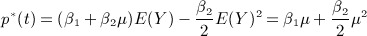
(12)

results in the same estimate as when applied to the clinical incidence, compare Equation (7).

## 6. Jointly estimating mean sojourn time and test sensitivity using maximum likelihood estimation

Up to now, we have skipped the problem of estimating prevalence in a situation with imperfect test sensitivity. We do not observe prevalence of preclinical disease directly; we only have observed detection rates *r*(*t*) = *p*(*t*) × *s*, where *s* is the sensitivity of the screening test. If we only have data for the detection rate at first screening and the background incidence, we can either equate the detection rate and the prevalence, effectively assuming 100% sensitivity as in 3,4, or use an independent estimate of the sensitivity from the literature as in 5. To estimate test sensitivity, we also need data on interval cancers diagnosed clinically after a negative screening test. We hope that interval cancer incidence can give us more insight in the difference between the catch-up time and the classic Markov models.

For the classic Markov model, the seminal papers by Zelen and Feinleib 1 and Day and Walter 2 describe how to jointly estimate mean sojourn time and test sensitivity using maximum likelihood estimation. Later, Shen and Zelen expanded these models to allow for incidence changing by age or time 8 and for nonparametric sojourn time distributions 9. We extend these methods to the catch-up time model in the simple setting of a single screening test, using data on background incidence, detection rate at screening, and incidence of interval cancers in successive years after the test. For comparison, we also present estimates for mammography screening for breast cancer based on data from the Health Insurance Plan (HIP) trial of Greater New York 10.

First, we need predictions for interval cancers from our models. Tumors diagnosed clinically at time *t* + *x* after a screening test at time *t* must have onset *Z* > *t* or have escaped detection. In the classic model of Section 5, we have


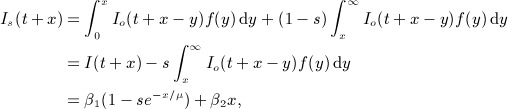
(13)

see for instance 8. For the catch-up time model, assuming the model from Section 3, we obtain for the incidence *I*_*s*_ after a screening test:


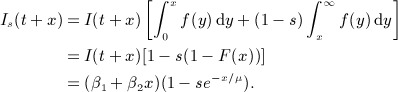
(14)

Like Day and Walter 2 and Zelen *et al.* 1, 8, we assume that observed numbers of cases detected at the screening test and interval cancers in the first years following the test are Poisson distributed with expected numbers calculated from Equations (6), (11), (13), and (14) and the relation among detection rate, prevalence, and sensitivity *R* = *s* × *P*. For breast cancer, we used published data from the HIP trial 2, 10, using 8, 11 to estimate the rate of increasing background incidence with time. For prostate cancer, we used data provided by the Rotterdam section of ERSPC (Table I). We calculated all estimates with the mle routine of R 12.

**Table I:** Maximum likelihood estimation of mean sojourn time and test sensitivity in breast cancer (HIP trial) and prostate cancer (ERSPC-Rotterdam): data summary and parameter estimates (Rates are expressed as the number of cases per 1000 person-years, except for the first-round detection rate, which is per 1000 persons)

	HIP trial		ERSPC	
		
Cases	*n*	Rate	*n*	Rate
Control arm	285	1.87	1067	5.26
First-round detection	55	2.73	1078	54
Interval cancers				
0–1 year	13	0.61	17	0.90
1–2 years	7	1.03	10	0.54
2–3 years	1	0.31	24	1.31
3–4 years	3	1.16	24	1.34
4–5 years	5	2.25	–	–

Parameter (standard error)	Catch-up model	Classic model	Catch-up model	Classic model
Background incidence per 1000, *β*_1_	1.75 (0.10)	1.75 (0.10)	3.57 (0.28)	3.72 (0.28)
Annual increase per 1000, *β*_2_	0.021 (0.00)	0.021 (0.00)	0.32 (0.05)	0.28 (0.05)
Mean sojourn time, *μ*	1.93 (0.54)	1.98 (0.57)	9.36 (0.45)	15.89 (1.45)
Sensitivity, *s*	0.81 (0.18)	0.81 (0.18)	0.89 (0.04)	0.92 (0.03)
Prevalence per 1000	3.36	3.36	61	59
Catch-up time	1.95	1.95	11.32	11.12
Mean lead time	1.98	1.98	13.58	15.89

Log-likelihood	− 17.436	− 17.437	− 65.708	− 67.744

Data for the HIP trial from 2,10; *β*_2_ fixed at 0.021/1000 per year 8,11.

Data for the ERSPC trial provided by Rotterdam trial center. Cut-off date for follow-up: February 1, 2008. Years used: years 1 through 12 since entry for incidence in the control arm, and years 1 through 4 since the first screening for interval cancers. Note that first-round detection rate is based on all detected cases and not only cases with PSA *≥*4 ng/mL as in 4.

Note that the first-round detection rate in the ERSPC data in Table I is higher than the rate in Figure 1. Finne *et al.* 4 limited the screen-detected cases to those with prostate-specific antigen (PSA) values larger than 4 ng/ml. In the Rotterdam trial, the protocol changed over time. At first, a PSA value larger than 4 ng/ml or a positive digital rectal exam (DRE) was considered a positive test; later, the PSA cut-off was lowered to 3 ng/ml, and the DRE test was dropped. Because of this and because of a less than 100% sensitivity, the mean sojourn time estimates obtained in Table I are larger than calculated from the data in Figure 1: 9.36 years versus 6.80 years in the catch-up time model and 15.64 years versus 11.64 years in the classic model. As before, the mean sojourn time estimate in the catch-up time model is approximately 60% of that in the classic model. In breast cancer screening, the mean sojourn time is much smaller, and the difference between the parameter estimates of the two models is negligible. For breast cancer, the log-likelihoods of both models are similar, but for prostate cancer, the log-likelihoods indicate a slightly better fit of the catch-up time model.

## 7. A closer look at the two models

**Figure 3:**
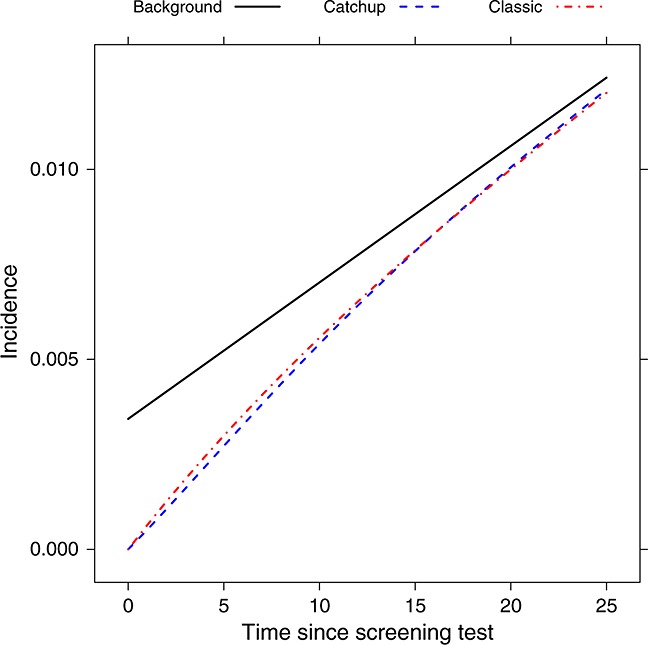
Prostate cancer incidence after a screening test with sensitivity 1.0 as predicted by the classic model (13) with *μ* = 11.64 and the catch-up time model (14) with mean sojourn time *μ* = 6.80 compared with background incidence without screening. Background incidence as given in Figure 1.

It is interesting to compare interval cancer incidence with background incidence, as the reduction of incidence due to the screening test provides a clue of where in time the cancers come from that are detected by screening. In Figure 3, we plotted the interval cancer incidence as computed from Equations (13) and (14). The predictions from the two models turn out to be remarkably similar, notwithstanding the different mean sojourn times.

Correspondingly, estimated mean lead time is more similar than expected from the widely different sojourn times. In the classic model with exponentially distributed sojourn times, lead time and sojourn time have the same distribution, and mean lead time equals mean sojourn time: 11.64 years in our numerical example. In our model for the catch-up time method with exponentially distributed sojourn times, a similar equivalence exists between the distributions of sojourn time and *time from onset Z* to time of screening *t*. That is, in prevalent cases at time *t*, onset occurred on average 6.8 years earlier. An estimate of mean lead time (time to clinical diagnosis *X*) may be obtained by evaluating mean sojourn time given being prevalent at time *t*. This conditional sojourn time is larger than the unconditional one (length biased sampling). In fact, in our numerical example, we have


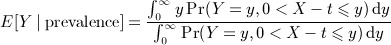
(15)


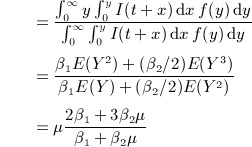
(16)

equal to 16.43 years. That is, mean lead time equals 16.43 − 6.80 = 9.63 years. Clearly, the estimates of mean lead time in the two models diverge less than the estimates of mean sojourn time. The same is seen in the models that were estimated using maximum likelihood (Table I).

## 8. Discussion

Screen-detected cancers are cancers that would have surfaced clinically at some time in the future, where this time depends on the sojourn time distribution. Intuitively, mean sojourn time is one of the determinants of catch-up time, that is, the time needed for background incidence to catch up with the detection rate at first screening.

In our note, we proposed a model to justify the catch-up time method more formally. Applying this model to the case of prostate cancer screening and comparing the results with classic Markov type models of Zelen and Feinleib and Day and Walter 1, 2, we found two things. First, in both models, the catch-up time method leads to biased estimates of mean sojourn time when background incidence is increasing with time. Second, the mean sojourn time estimates based on our catch-up time model differ considerably from estimates based on the classic Markov-type models. The differences in the estimates are a consequence of the presence of a trend in background incidence; without such a trend all estimates are equal.

Assuming independence between the time of *clinical incidence* and sojourn time as in our catch-up time model, we showed that the catch-up time method yields estimates of mean sojourn time biased toward higher values when incidence is increasing with time. Similarly, in the classic Markovian models, assuming independence between the time of *onset* and sojourn time, the catch-up time method results in estimates biased toward lower values, when incidence is increasing with time. In our numerical example, with linearly increasing incidence and exponentially distributed sojourn times, we obtained three estimates of mean sojourn time: 6.80 < 8.16 < 11.64, all based on the same data 4.

Applying the maximum likelihood methods proposed in the classic papers on breast cancer screening 1, 2 confirmed our direct calculations. For prostate cancer, the two models result in different estimates of mean sojourn time: 9.36 years in the catch-up time model and 15.89 in the classic model. For mammography screening for breast cancer in the HIP trial, the estimates from both models are almost equal: 1.93 and 1.98 years, respectively.

Why are the mean sojourn times so different in the two models? Because of the different independence assumptions, future incidence enters explicitly in the expression for prevalence in our catch-up time model (Equations (6) and (11)). In the classic models with exponentially distributed sojourn times, prevalence of preclinical disease does depend only on incidence at the time of screening (*β*_1_*μ*) and not on future incidence, as was already demonstrated by Zelen and Feinleib 1. In our catch-up time model, however, the future trend enters explicitly in the prevalence equation (*μ*(*β*_1_ + *β*_2_*μ*)), showing the difference to be proportional to the product of trend and mean sojourn time. This is reflected in the maximum likelihood estimates of sojourn time in Table I: nearly equal in the case of breast cancer and substantially different in the case of prostate cancer, where the trend is approximately 10 times larger and the estimated mean sojourn time is eight times larger.

It is interesting to compare the range above with the statistical confidence intervals of the estimates. Assuming a Poisson distribution for the number of men detected at the first screen, 804 in the example data of Figure 1, gives the following 95% confidence intervals for the mean sojourn time (in years): 6.80 (6.48–7.13), 8.16 (7.72–8.59), and 11.64 (10.64–12.45). Here, we neglected the variability in the background incidence, which was taken into account in our maximum likelihood estimates. The maximum likelihood estimates and confidence intervals were 9.36 (8.48–10.24) for the catch-up time model and 15.89 (13.05–18.73) for the classic model. The message remains that uncertainty about the model is greater than the statistical uncertainty.

In contrast to the difference in sojourn time, the models show better agreement on the estimated lead time. The reduction of interval cancer incidence compared with background incidence at different times after the screening test indicates where in time the screen-detected tumors are expected to surface clinically in the absence of screening. Figure 3 shows that the predicted interval cancer incidence is very similar in the two models.

Which of the two models is more appropriate is difficult to say, as sojourn time and lead time are never observed. In any case, the differences in the log-likelihoods obtained in Table I are too small to make a choice between the models. The classic models of Zelen and Feinleib and Walter and Day might seem more natural if we think of tumors becoming preclinical and clinical as biological events in time. However, we must realize that the onset of preclinical disease is not simply a biological event (the start of a tumor) but an abstract event depending on tumor development and (changing) diagnostic technology. An illustration of this is provided by the difference in sojourn time estimates as estimated from Figure 1 and the estimates in Table I. Finne *et al.* 4 only considered screen-detected tumors with PSA 4 ng/ml to make estimates comparable across ERSPC centers. The actual screening test in Rotterdam, on which the data in Table I are based, used a PSA cut-off level of 3 ng/ml for most screenings (and included digital rectal examination for the remaining screenings). The higher cut-off value for PSA considered by Finne *et al.* together with the assumed 100% test sensitivity causes a decrease of mean sojourn time from 9.36 to 6.80 years in the catch-up time model and from 15.89 to 11.64 years in the classic model. Similarly, the event of clinical diagnosis depends on both biology and diagnostic technology. So, it might not be so clear that assuming independence between onset and sojourn time is a better model than assuming independence between sojourn time and clinical incidence.

In any case, the independence assumption is just one of the assumptions we make in order to estimate quantities that we cannot observe directly. Another convenience assumption was to assume sojourn times to be exponentially distributed; it would be worthwhile to investigate whether our numerical results would be different with other distributions.

Our attempt to find a formal justification of the catch-up time method failed: The catch-up time method does not estimate sojourn time or lead time correctly in either model we considered. Therefore, we hope that our note has demonstrated the importance of making assumptions explicit and that estimates cannot be simply transferred from one model to another.
